# Supplementary material for: Polarity protein SCRIB interacts with SLC3A2 to regulate proliferation and tamoxifen resistance in ER+ breast cancer
Source: Commun Biol. 2022 May 2;5:403. doi: 10.1038/s42003-022-03363-3 (PMC9061724; doi:10.1038/s42003-022-03363-3)

Supplementary Information

Polarity protein SCRIB interacts with SLC3A2 to regulate proliferation and tamoxifen resistance in ER+ breast cancer

Yasuhiro Saito[1*], Shiori Matsuda[1], Naomi Ohnishi[2], Keiko Endo[1], Sanae Ashitani[1], Maki Ohishi[1], Ayano Ueno[1], Masaru Tomita[1], Koji Ueda[2], Tomoyoshi Soga[1*], Senthil K. Muthuswamy[3*]

[1]Institute for Advanced Biosciences, Keio University, 246-2 Mizukami, Kakuganji, Tsuruoka, Yamagata, 997-0052, Japan.

[2]Cancer Proteomics Group, Cancer Precision Medicine Center, Japanese Foundation for Cancer Research, 3-8-31, Ariake, Koto, Tokyo, Japan.

[3]Department of Medicine and Pathology, Cancer Research Institute, Beth Israel Deaconess Medical Center, Harvard Medical School, 3 Blackfan Circle Boston MA, 02215, USA

Supplementary Information includes:

Supplementary Figure 1-7

Raw data of immunoblot and gel images

Supplementary data 1-2 (Excel file)

Supplementary Figure 1

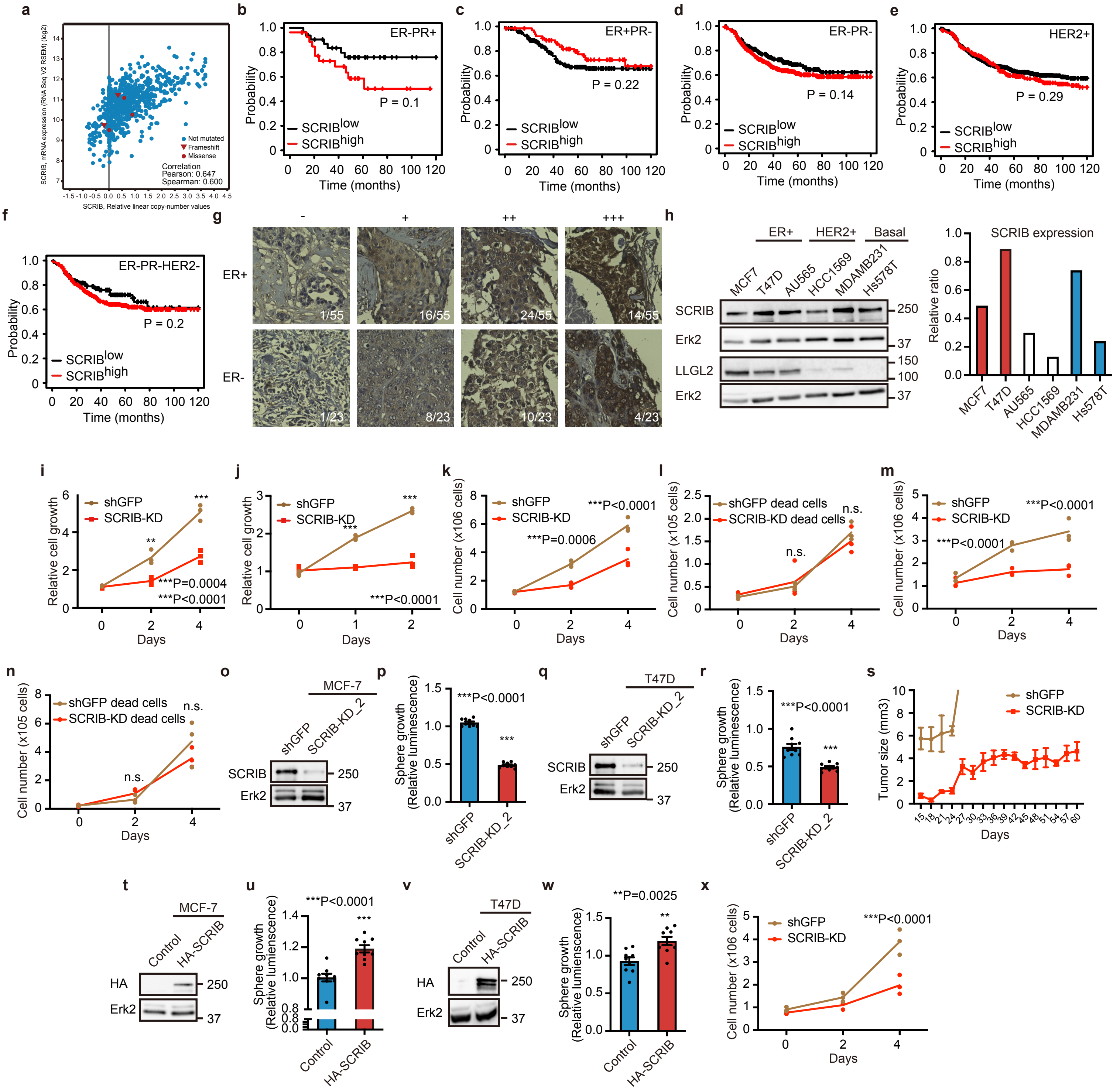

Supplementary Figure 1 | SCRIB promotes cell proliferation in ER+ BC cells. a, Correlation between copy number variation of SCRIB gene and mRNA expression in breast cancer data from The Cancer Genome Atlas (TCGA). b-f, Kaplan-Meier plot of ER-PR+ (b), ER+PR- (c), ER-PR- (d), HER2+ (e), and ER-PR-HER2- (f) breast cancer patients' survival. g, Immunohistological images of SCRIB protein in ER+ and ER- breast cancer tissues. h, Total protein levels of SCRIB and LLGL2 in ER+, HER2+, and basal breast cancer cell lines. Immunoblot images (left) and the relative ratio of signal intensities are shown (right). i, j, 2D growth of SCRIB-KD MCF-7 (i) and T47D (j) cells. k-n, Changes in live (k, m) and dead (l, n) cell number of MCF-7 (k, l) and T47D (m, n) cells. o, Knockdown of SCRIB in MCF-7 cells. p, Cell viability assay in SCRIB-KD MCF-7 cells. q, Knockdown of SCRIB in T47D cells. r, Cell viability assay in SCRIB-KD T47D cells. s, Magnified graph of tumor growth of SCRIB-KD MCF-7 cells shown in Fig. 1f. t, Overexpression of HA-tagged SCRIB in MCF-7 cells. u, Cell viability assay in HA-SCRIB expressing MCF-7 cells. v, Overexpression of HA-tagged SCRIB in T47D cells. w, Cell viability assay in HA-SCRIB expressing T47D cells. x, 2D growth of SCRIB-KD MCF-7 cells in growth medium supplemented with 10% FBS. Data p, r, s, u, and w are shown as mean ± s.e.m.; b; n=59, c; n=248, d; n=677, e; n=882, f; n=534, i, j, k, l, m, n, and x; n=3, p, r, u, and w; n=9. Statistical analysis was conducted by t-test (p, r, u, w) and two-way ANOVA followed by Tukey's posttest (i, j, k, l, m, n, x).

Supplementary Figure 2

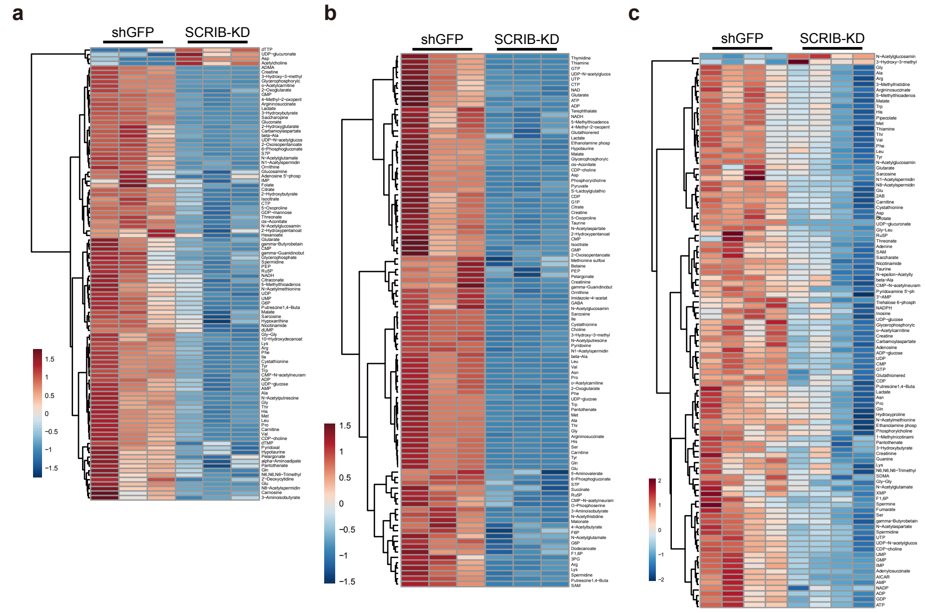

d

Downregulated metabolites
GMP
Glycerophosphorylcholine
Creatine
o-Acetylcarnitine
UDP-N-acetylglucosamine
N1-Acetylspermidine
Cystathionine
CMP-N-acetylneuraminate
N-Acetylglucosamine 1-phosphate
Tyr
Arg
Gly
Asp
Carnitine
Threonate
Leu
Carbamoylaspartate
ADP
CDP-choline
UDP-glucose
Glu
AMP
UDP
CMP
Malate

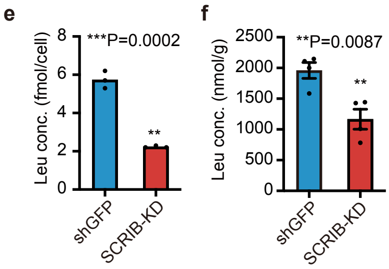

Supplementary Figure 2 | Metabolome analysis in SCRIB-KD ER+ breast cancer cells. a, b, c, Heatmap of metabolites in SCRIB-KD MCF-7 (a), T47D (b) cells, and mouse tumors (c). d, List of metabolites downregulated in SCRIB-KD cells. e, f, Relative concentration of intracellular leucine in T47D cells (e) and tumors (f). Data e and f are shown as mean ± s.e.m.; e and f; n=3. Statistical analysis was conducted by t-test (e, f).

Supplementary Figure 3

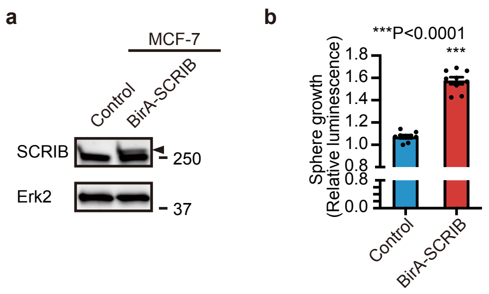

c

Gene name	Abundance ratio (SCRIB/Control)
VIM Vimentin	1000
GIT1 ARF GTPase-activating protein	1000
ITGA5 Integrin alpha-5	1000
CTNNB1 Catenin beta-1	1000
LLGL2 LLGL scribble cell polarity complex component 2	1000
NOS1AP Carboxyl-terminal PDZ ligand of neuronal nitric oxide synthase protein	1000
SCRIB Protein scribble homolog	77.875
ACTN1 Appha-actinin-1	57.594
ACTN4 Appha-actinin-4	49.441
TJP1 Tight junction protein ZO-1	24.694
VANGL1 Vang-like protein 1	19.164
TJP2 Tight junction protein ZO-2	18.927
DLG5 Disks large homolog 5	8.94
DLG1 Disks large homolog 1	8.9
SLC7A5 Large neutral amino acids transporter small subunit 1	5.901
CDH1 Cadherin-1	2.9989
RAC1 Ras-related C3 botulinum toxin substrate 1	1.781
YWHAG 14-3-3 protein gamma	1.614
SLC3A2 4F2 cell-surface antigen heavy chain	1.584
SUGT1 Protein SGT1 homolog	1.567
CTNND2 Catenin delta-2	1.561
CTNND1 Catenin delta-1	1.530
TRIP6 Thyroid receptor-interacting protein 6	1.216
AP2B1 AP-2 complex subunit beta	1.176
AP2A1 AP-2 complex subunit alpha-1	1.168
ARVCF Armadillo repeat protein deleted in velo-cardio-facial syndrome	1.004
CFL1 Cofilin-1	0.855
KRT18 Keratin, type I cytoskeletal 18	0.78

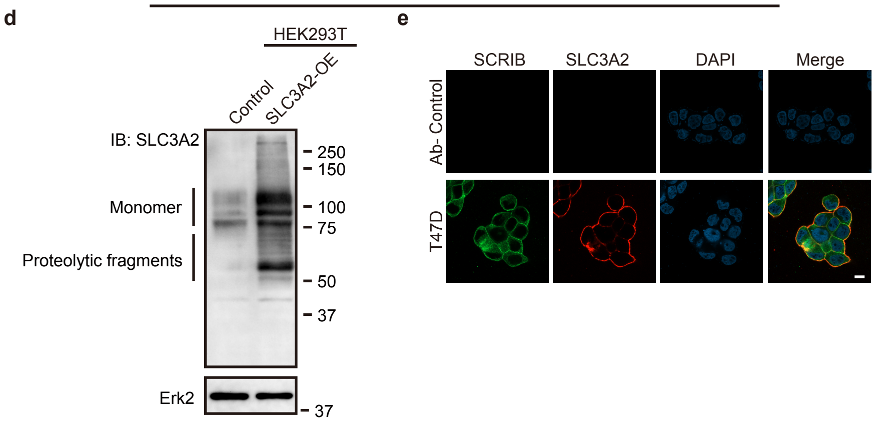

Supplementary Figure 3 | SCRIB interacts with SLC3A2 in ER+ breast cancer cells. a, Overexpression of BirA*-SCRIB in MCF-7 cells. Arrowhead indicates the position of BirA*-SCRIB. b, Cell viability assay in BirA*-SCRIB-expressing MCF-7 cells. c, List of SCRIB-binding proteins identified by BioID. d, Validation of anti-SLC3A2 antibody. The cell lysates from HEK293T cells expressing SLC3A2 were immunoblotted with anti-SLC3A2 antibody. e, Immunostaining images of SCRIB and SLC3A2 in T47D cells. Scale bar, 10 μm. Data b is shown as mean ± s.e.m.; b; n=9. Statistical analysis was conducted by t-test (b).

Supplementary Figure 4

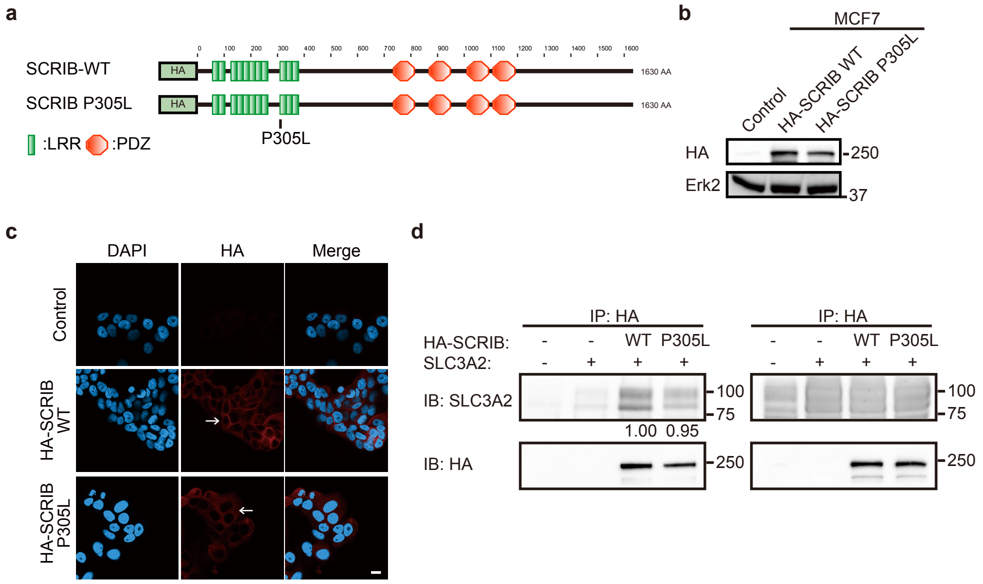

Supplementary Figure 4 | SCRIB P305L mutant interacts with SLC3A2. a, Cartoon representation of wild-type SCRIB and SCRIB P305L mutant. b, Overexpression of HA-tagged SCRIB and HA-tagged SCRIB P305L in MCF-7 cells. c, Immunostaining images of HA-tagged SCRIB and HA-tagged SCRIB P305L mutant in MCF-7 cells. Scale bar, 10 μm. d, Immunoprecipitation of HA-tagged wild-type SCRIB or SCRIB P305L mutant in HEK293T cells.

Supplementary Figure 5

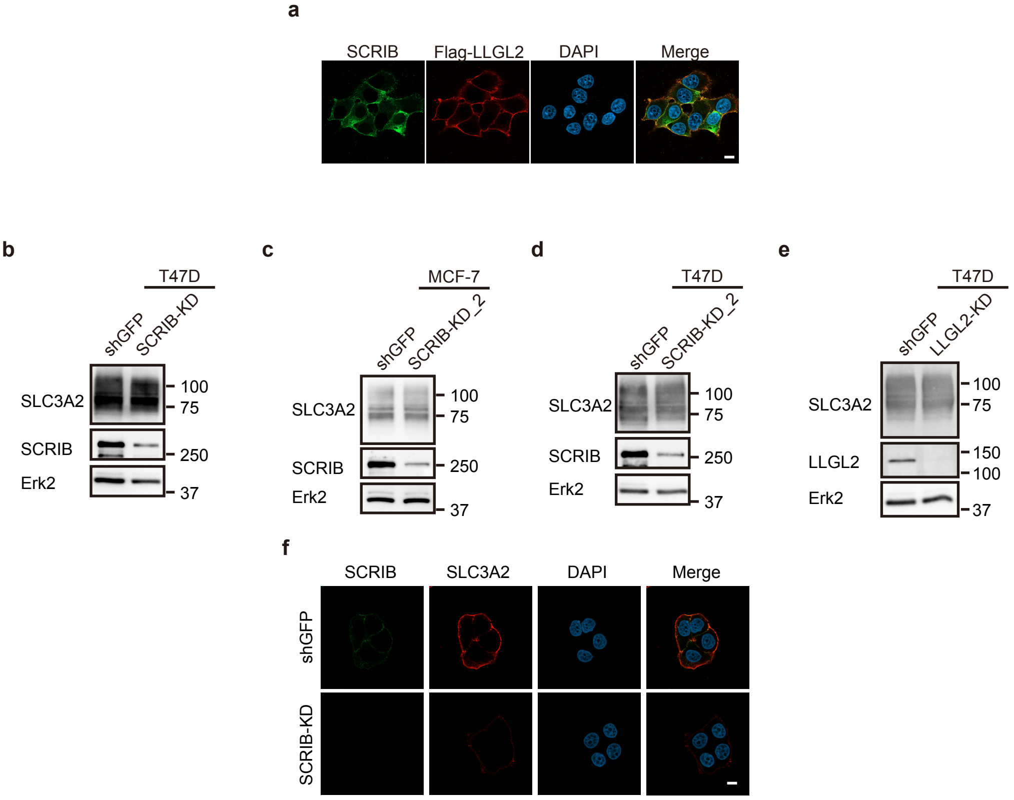

Supplementary Figure 5 | SCRIB regulates membranous localization of SLC3A2 in ER+ breast cancer cells. a, Co-localization of SCRIB and LLGL2 in MCF-7 cells. b, Total protein levels of SLC3A2 in SCRIB-KD T47D cells. c, Total protein levels of SLC3A2 in SCRIB-KD MCF-7 cells. d, Total protein levels of SLC3A2 in SCRIB-KD T47D cells. e, Total protein levels of SLC3A2 in LLGL2-KD T47D cells. f, Immunostaining images of SCRIB and SLC3A2 in T47D cells. Scale bar, 10 μm.

Supplementary Figure 6

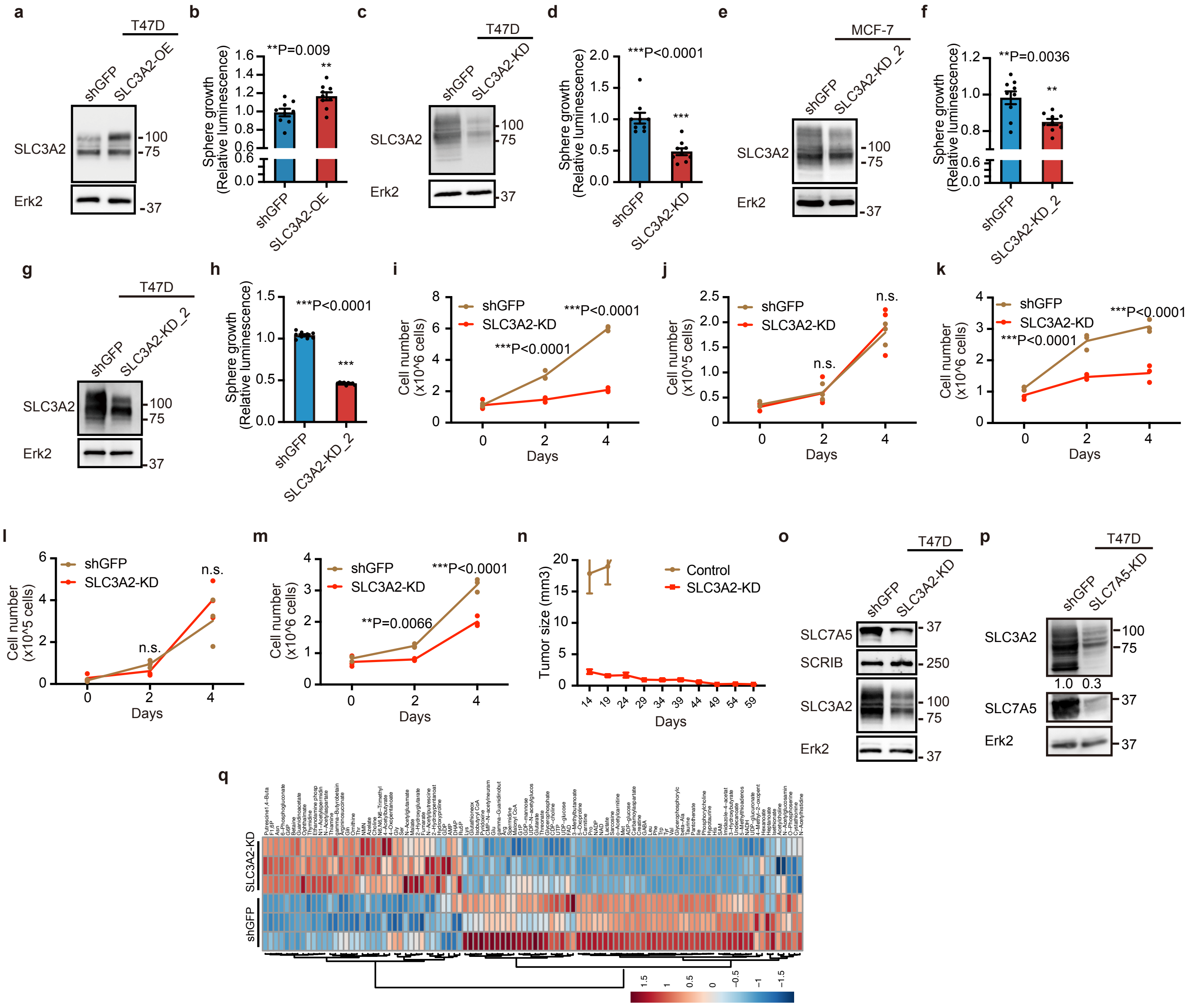

Supplementary Figure 6 | SLC3A2 promotes cell proliferation in ER+ breast cancer cells. a, Overexpression of SLC3A2 in T47D cells. b, Cell viability assay of SLC3A2-OE T47D cells. c, Knockdown of SLC3A2 in T47D cells. d, Cell viability assay of SLC3A2-KD T47D cells. e, Knockdown of SLC3A2 in MCF-7 cells. f, Cell viability assay of SLC3A2-KD MCF-7 cells. g, Knockdown of SLC3A2 in T47D cells. h, Cell viability assay of SLC3A2-KD T47D cells. i-l, Changes in the number of live (i, k) and dead (j, l) cells in MCF-7 (i, j) and T47D (k, l). m, 2D growth of SLC3A2-KD MCF-7 cells in the culture medium supplemented with 10% FBS. n, Magnified graph shown in Fig. 4g. o, Total protein levels of SLC7A5 in SLC3A2-KD T47D cells. p, Total protein levels of SLC3A2 in SLC7A5-KD T47D cells. q, Heatmap of metabolites in SLC3A2-KD T47D cells. Data b, d, f, and h are shown as mean ± s.e.m.; b, d, f, h; n=9, i, j, k, l, m; n=3. Statistical analysis was conducted by t-test (b, d, f, h) and two-way ANOVA followed by Tukey' s posttest (i, j, k, l, m).

Supplementary Figure 7

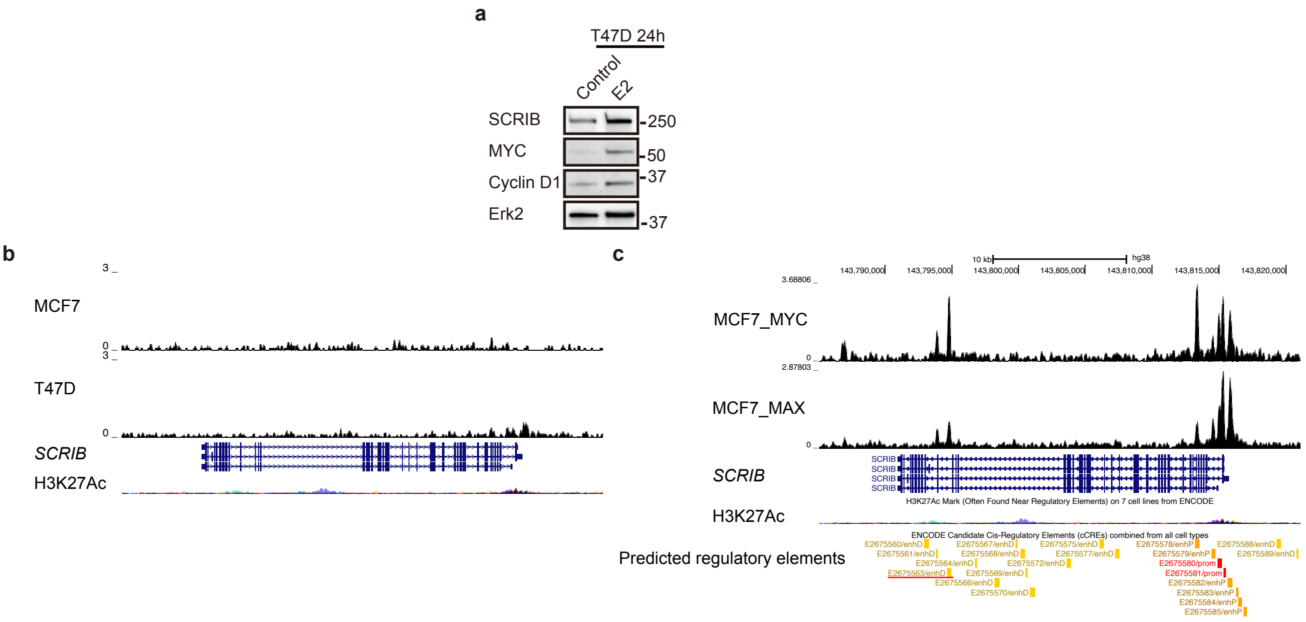

d

chr8: 143795583-143796783

PCR primer (forward): AGCCCAGAATAGTCAGCAAC

CTCTGGGGCAGGCTGGGGTCCCAGCCCAGAATAGTCAGCAACGGTGAGCCCAGGAGCCGCGTCCCTGGGCAGGGCTGACCGCGTGGCTGGGGCAGCCTTGGAGGGCACTCCGCTGGGCCCCCCAG
GACGCTGCAGCACTGTCCCCGGCCGGTCCCCATCACAGCTCACAGGGCTGCCGTGGACGTGGTCACGTCTGCACTAGGCAAGACTGTGAAGTAGCATGATGCCTGCCACTCTGCTGACTGTGTAA
AGTCATCCCCAGGAAGAGCTGACAGCCACCAAGATGGGGGAAGGAGGGGCGTGAGTAGCAGGCACTGGAACTGATGGGGCAGAACGGCCCCCTCGGGTGGAAAGGCTGGCTCCAGACCCCAGTGT

gRNA: CCTCGGGTGGAAAGGCTGG

GAGTGCTGGGCAGGGATTCTGAAACAAGGGACACCATGCAGGAGGCCCGGCCAGCTCTGCACTCCCCAGAGCTCAATCACCTGTCCCAAGCCAGAGACTGGCAGGGCGGAGGGGGCGTCTAGAAA
GGAAAGGCACTCAGATAGCTCAGGCAGGGCAGAGGGGGGCGAGGAGACAGGGCCAGGACAGGCAGGGGCTTCTGGTTGGGCACAAGATGTACTTGAATCCCGAGCCTGGGGTTAACACGGGCAGA
GGGAACCATGGGAAGAGCTGACTTCCAAGCCTGCGGCGGGCAACGGGACTGCTTCAAACCTCCACCCCCAGCAGCAGCCAGAAGCAGCGGAGGAGGCAGCACAGTGGACACAGGCATTGCTCACC
GCCCTCATCACAGACGGGGTCCCTGCAAAATCGAGGAGCTCTCAGTGTGGCCTCAGGGACAAACCCAGCCCTGTACTGTTGGTTAAAAACACAGCTGAAATGGTAAGTAAGGGGCAGGCAAAGAG
ATGCCAGGCAAACACACGGGAGGCACAGGGAGGGGCTGCTCAGGCCGGGGGAGAAAGGGTGTGAGTTCAGAGCTGTCGAGCAGGCAGACGGATGCCGTCACCACAGAAGATGCTGTTTACAACGA

gRNA: TGCCAGGCAAACACACGGG

TGATACAGTCATCAACTGTAAGCGCTCAACAAGGAAGCAGCTAAACACAGAAAGCAAAGACTAGAAACGCGAGTGTCGTAAAAGCACAGTTACACAGGCAGACTTAGCTACGTCTCTTTCAGACT
CACAGGACTAGCGGGACAAACAACAAACAAGGTGCCTGTCTTCTCAGTTTCTGAAATAACAAGTCAAAAAGCCAC

PCR primer (reverse): ACTTAGCTACGTCTCTTTCA

e

chr8: 143794345-143795219 (enhancer region: chr8:143794647-143794982)

PCR primer (forward): TACCCAAGAGCCCCCGAAT

TGCCCCCATGACTGTGCTGTTGTTCATGAGGATGAAGGGCCTCCCGGAAGCCTCCCTCTTGGCGCTGCCCATGGCCAGAGGAGGCAGGGTCAGTACCCAAGAGCCCCCGAATGCGCTGCCTCAGA
TCCAGGCCCTGCCTGGCCCCTCACCACCACCCCACCGCTTGGGCAAGGGCCTGCAGGAGCCACCCCTCGGCACTCACGTTCGGTGGGGCTGGTGGGGCGCACCCTCTGGAGGGTTATTTGTATC
TCTAAGGGGAGGGATGGGCCTGCACATCCTCCTGCCTGTCCCCAGCACCCGCCCCTCCAGCTGCTCCAGCCTGTCCTCCCCTTGGGGACATGTGCCTGCCCAATCCCCCAGGAGCCTCTGCCCCC

gRNA: CCCCTTGGGGACATGTGCC

ACTCGCACCCTCTGGGGCTGGGCAGCCCCAGCAGCAGCAGGTGAAGCTCTCGCACCGCCACTAGAGGGATGCCTCGGCCTGTGTCACCCATCCGCAGCTCAGGGTGTGTGGGTGGGGCAGCACCA
ACATGGCTTGGGTTGAGAATGAGGGTGATAAGCATGTGCCAGGCTGGGGTAGCCTGCAGGTCAGACGTGGCCTCCTCCCACCCCAGCCCCCGCCCAAAGGTTCCCTCCCGGATGGCCCTGGCGTT

gRNA: GTAGCCTGCAGGTCAGACG

TGTCCTGGGGTGGCCGATGAACAGGACAACAGGACGGAGGTGGGGGCACTGCACACTGGGCACTCACCCTCTGCACGCTGCCAGCGCTGGGCACGGCGGCCAGGGCGCGGTAGTCCAGCTTCAG

PCR primer (reverse): CCGATGAACAGGACAACAGG

GGGCTGCAGACCCCGACCCTGGGGGCAGAGTGAACACAGCACTAGCAGGGGTAGCTCCGTGGCAGCACCCGGCCAGCACAGGGCAGGAGAGGGGGTCCTGGGGGTTACTACCCAGCACCCCACAG

Supplementary Figure 7 | SCRIB expression is regulated by MYC in ER+ BC cells. a, Induction of SCRIB by E2 stimulation. b, ChIP-Seq data of ER in MCF-7 and T47D cells. c, ChIP-Seq data of MYC and MAX in MCF-7 cells. The predicted promoter or enhancer regions are shown. d, Genomic sequence of the chr8: 143795583-143796783 region. Primers for PCR and two guide RNAs for CRISPR system are described. e, Genomic sequence of the chr8: 143794345-143795219 region. Primers for PCR and two guide RNAs for CRISPR system are described.

Figure 1b

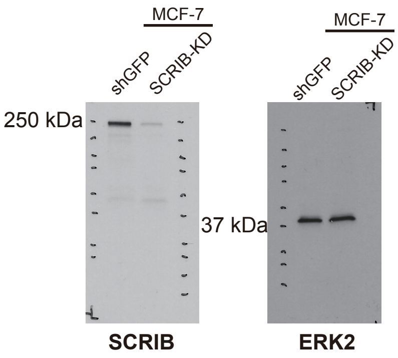

Figure 1d

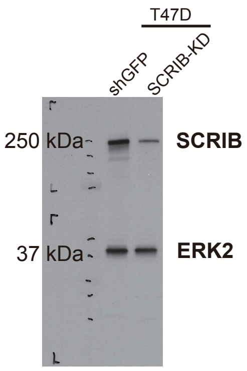

Figure 2a

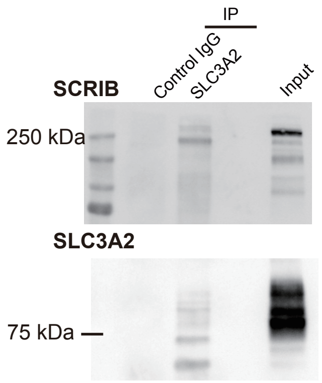

Figure 2c

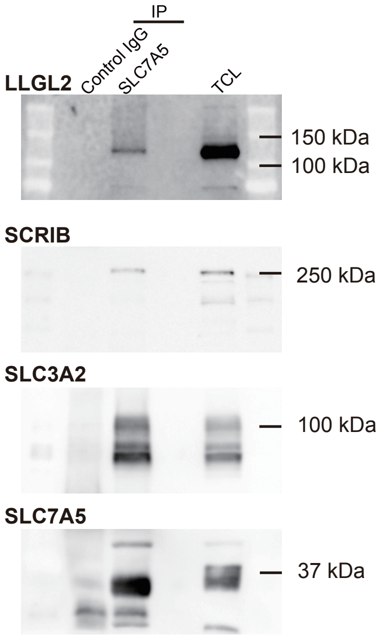

Figure 2d

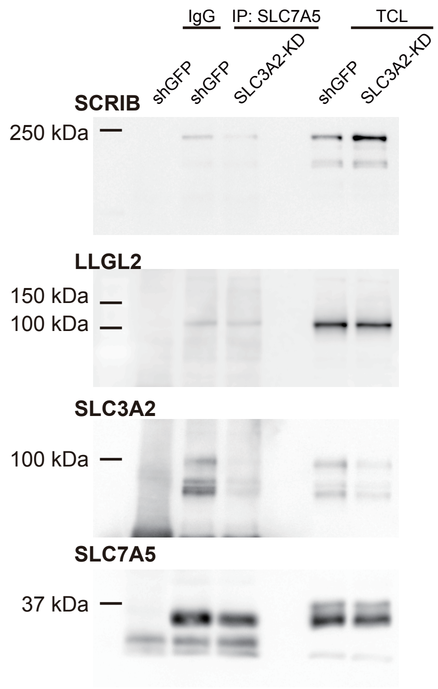

Figure 2e

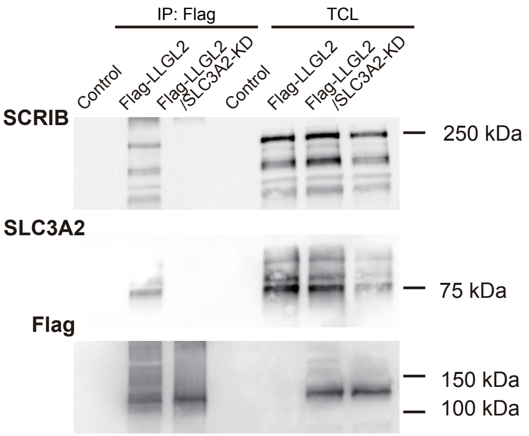

Figure 2g

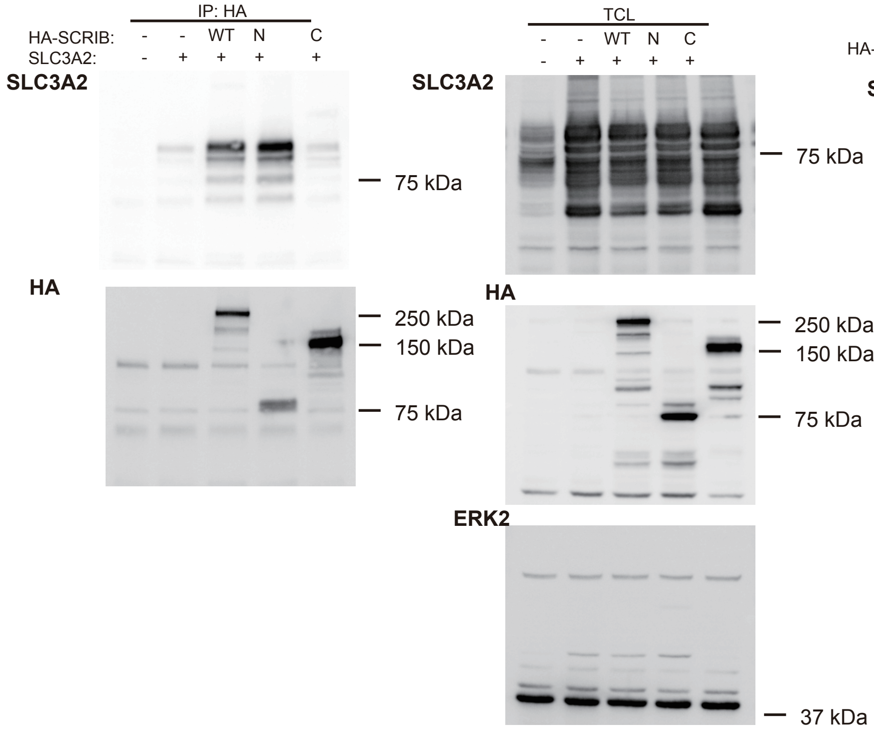

Figure 2h

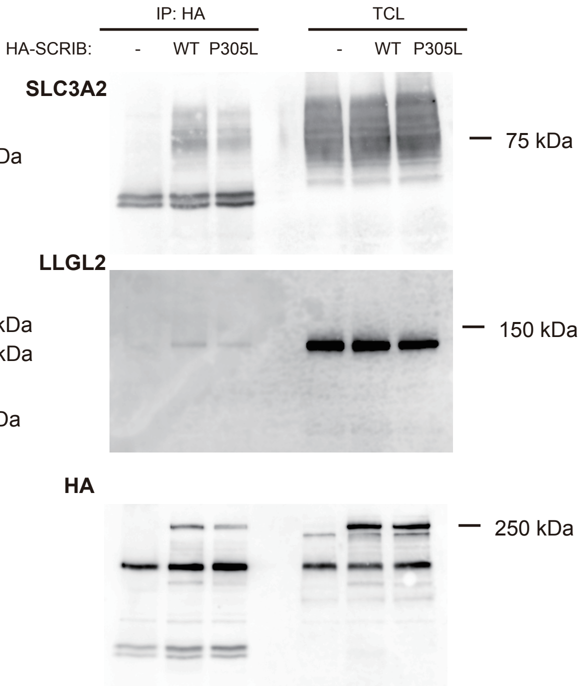

Figure 3a

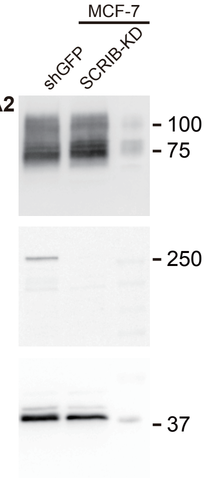

Figure 3b

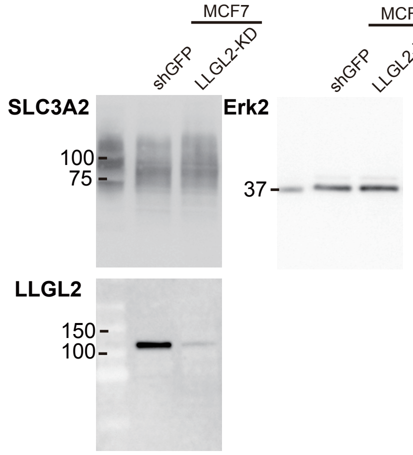

Figure 3e

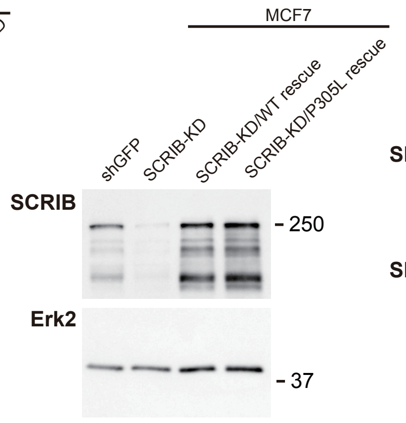

Figure 4b

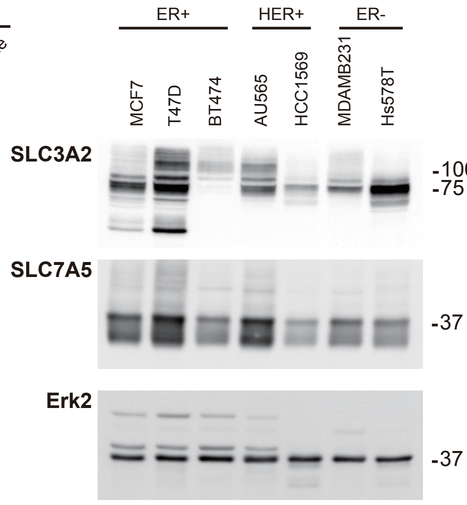

Figure 4c

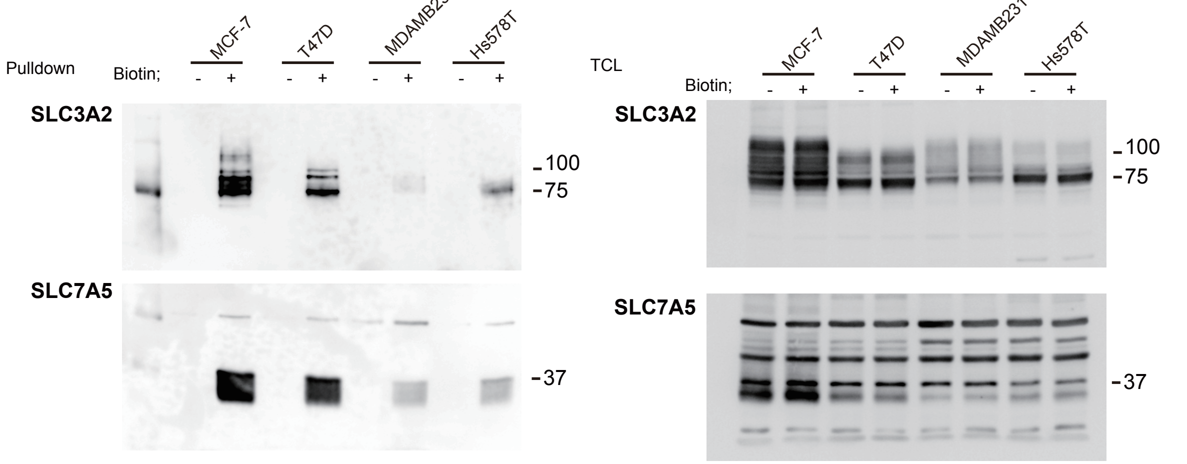

Figure 4e

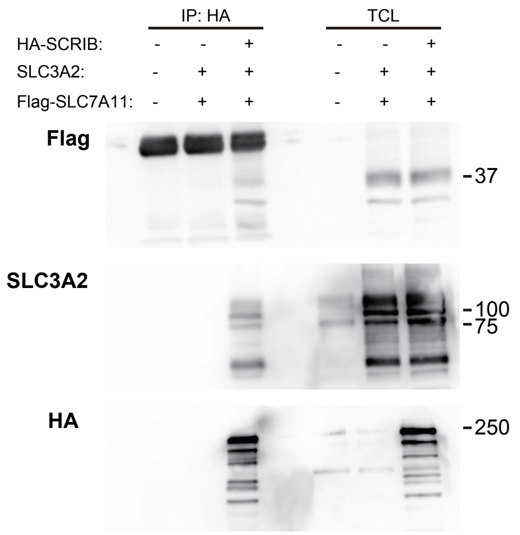

Figure 4f

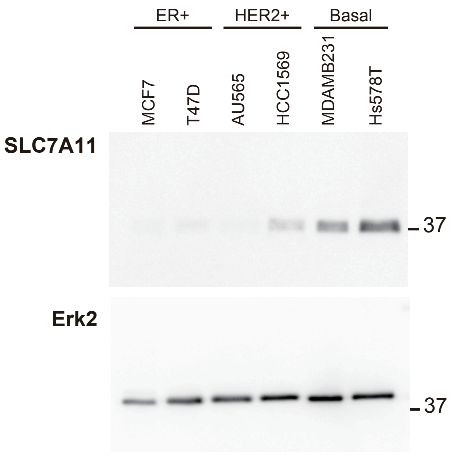

Figure 5b

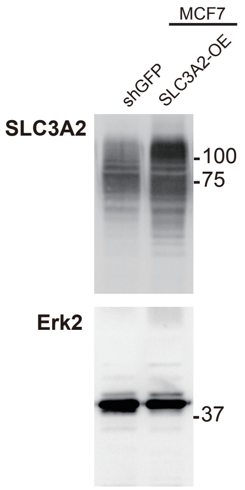

Figure 5d

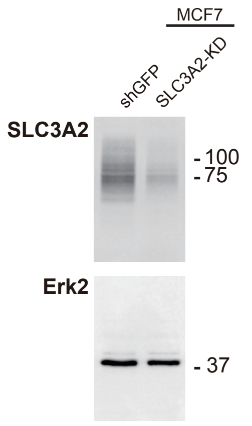

Figure 5h

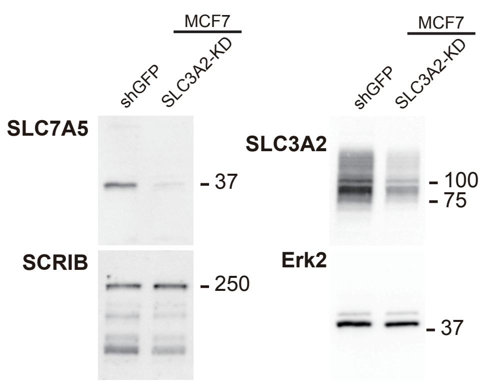

Figure 5i

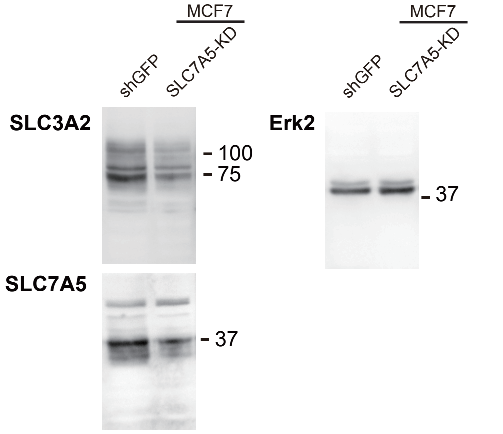

Figure 6a

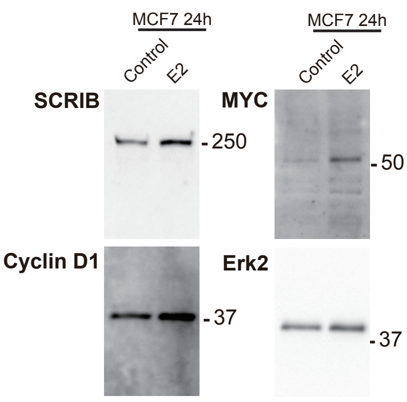

Figure 6c

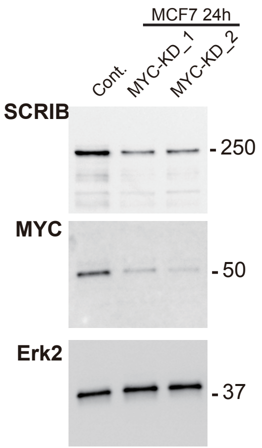

Figure 6d

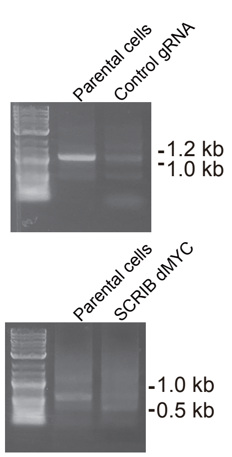

Figure 6e

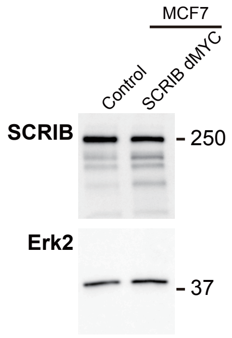

Figure 7b

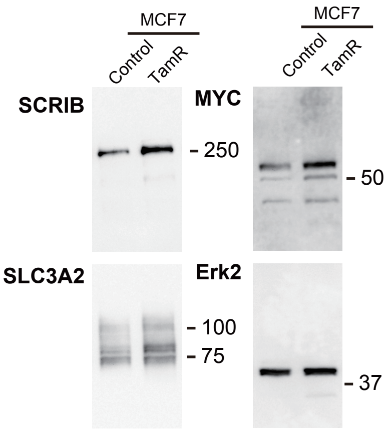

Figure 7c

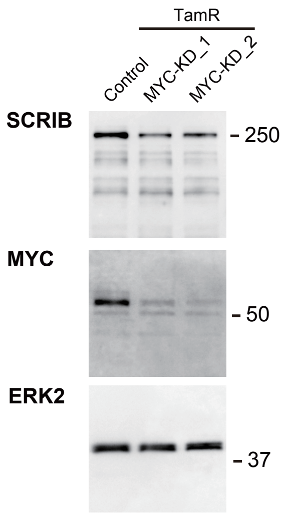

Figure 7d

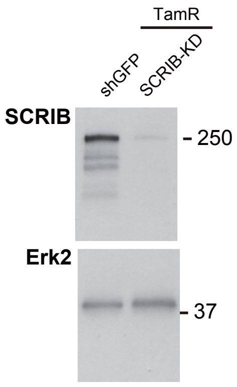

Figure 7f

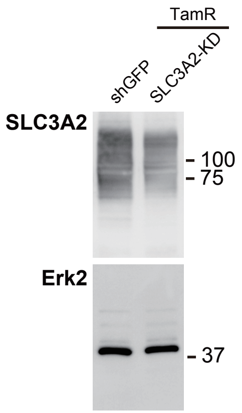

Supplementary Fig. 1h

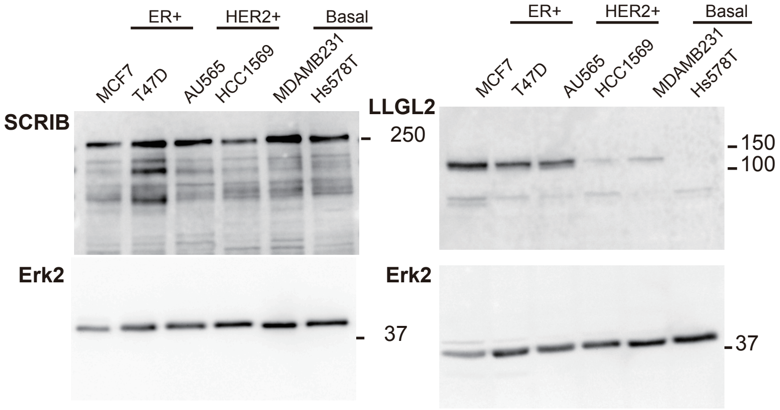

Supplementary Fig. 1o

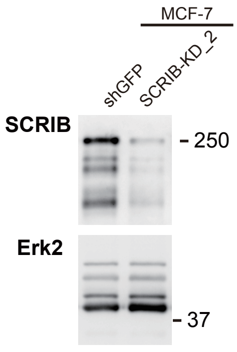

Supplementary Fig. 1q

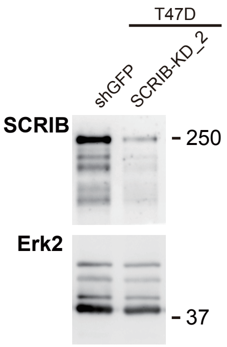

Supplementary Fig. 1t

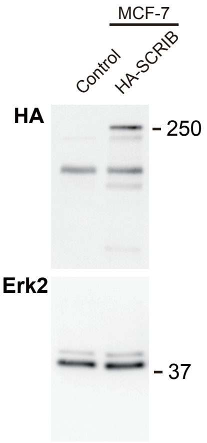

Supplementary Fig. 1v

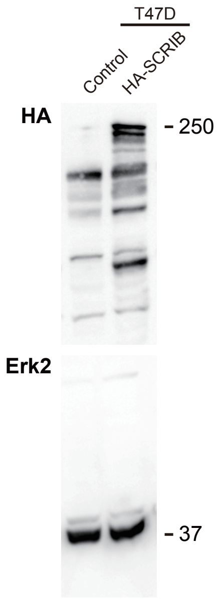

Supplementary Fig. 3a

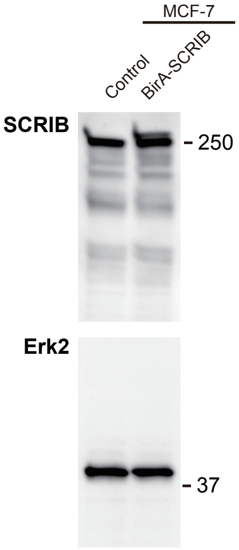

Supplementary Fig. 3d

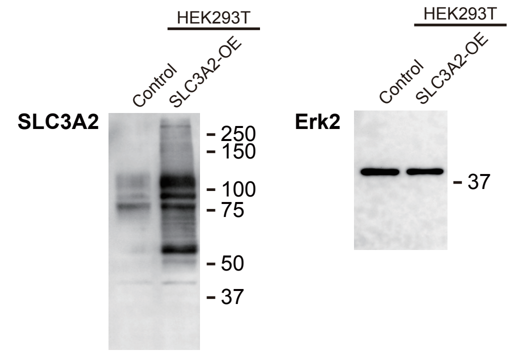

Supplementary Fig. 4b

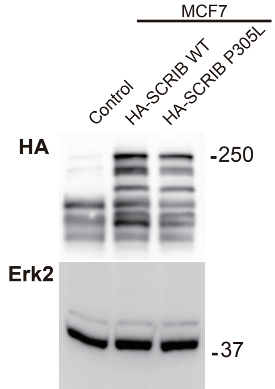

Supplementary Fig. 4d

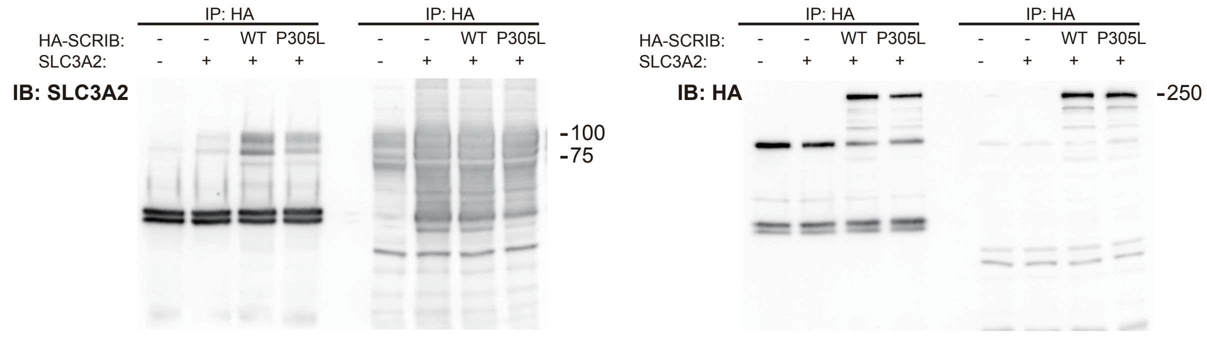

Supplementary Fig. 5b

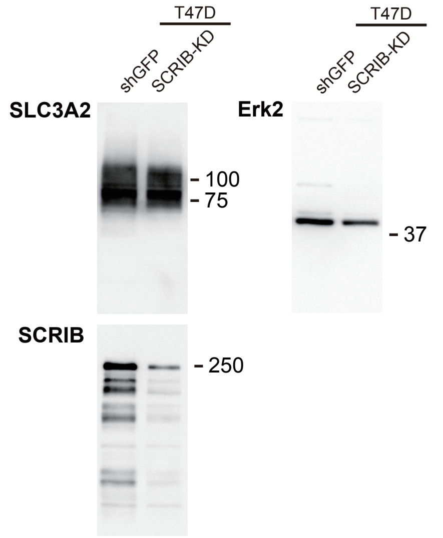

Supplementary Fig. 5c

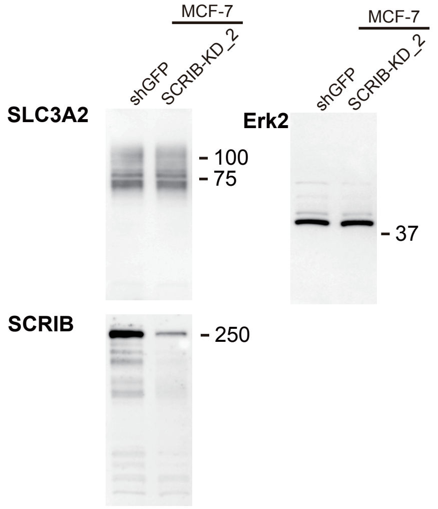

Supplementary Fig. 5d

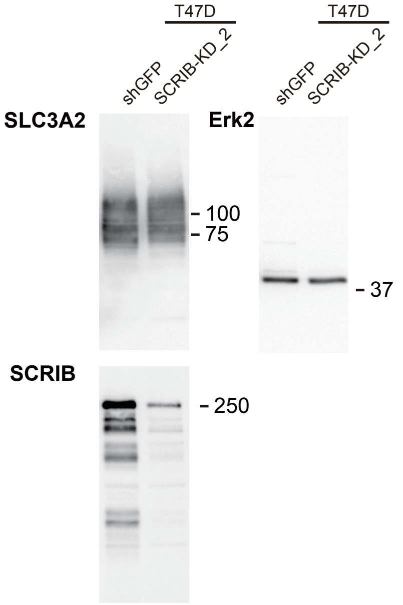

Supplementary Fig. 5e

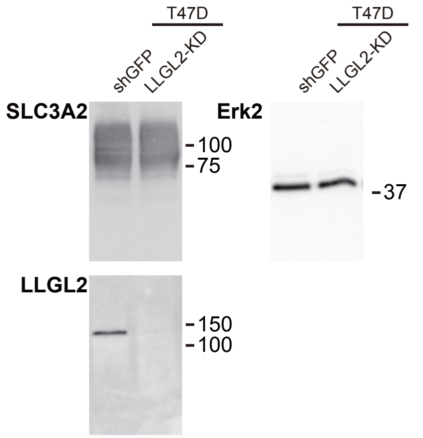

Supplementary Fig. 6a

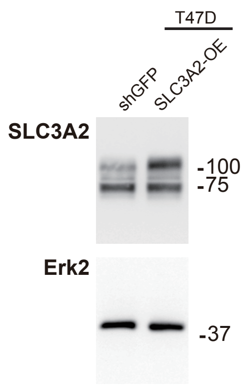

Supplementary Fig. 6c

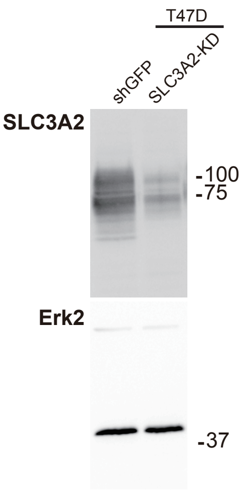

Supplementary Fig. 6e

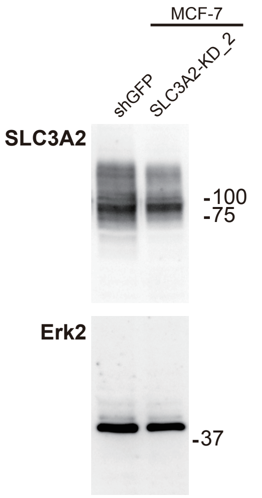

Supplementary Fig. 6g

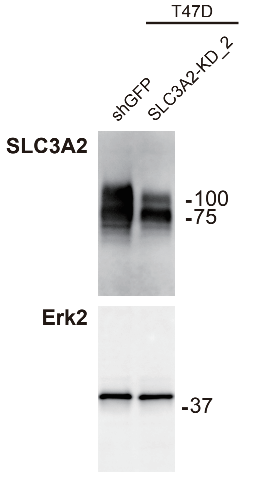

Supplementary Fig. 6o

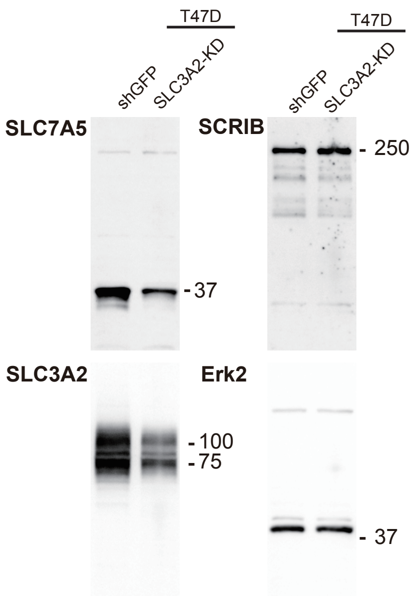

Supplementary Fig. 6p

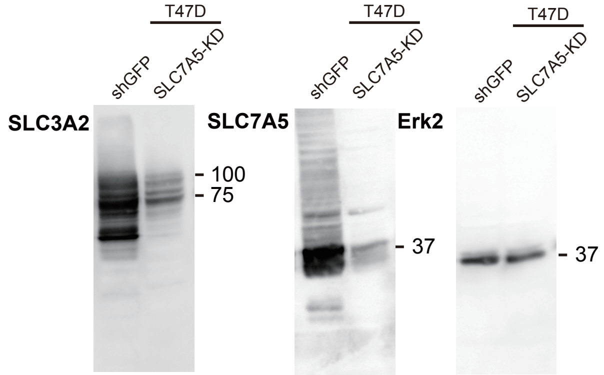

Supplementary Fig. 7a

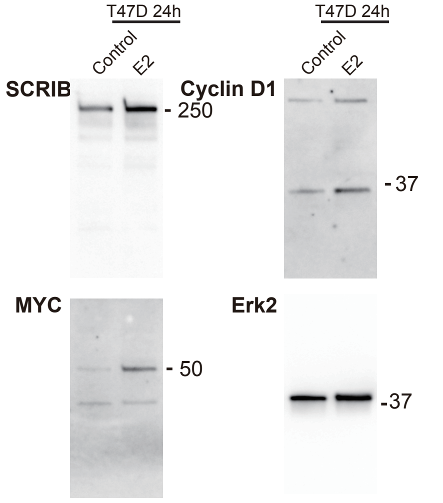

Supplement: Supplementary file 2 — Supplementary Information [file 42003_2022_3363_MOESM2_ESM.pdf]
