# Supplementary material for: Polarity protein SCRIB interacts with SLC3A2 to regulate proliferation and tamoxifen resistance in ER+ breast cancer
Source: Commun Biol. 2022 May 2;5:403. doi: 10.1038/s42003-022-03363-3 (PMC9061724; doi:10.1038/s42003-022-03363-3)
Supplement: Supplementary file 3 — Description of Additional Supplementary Files [file 42003_2022_3363_MOESM3_ESM.pdf]

Description of Additional Supplementary Files

File name: Supplementary Data 1
Description: Source data for Figure 1-5.

File name: Supplementary Data 2
Description: Source data for Supplementary Fig. 1-7.
