# Supplementary material for: Polarity protein SCRIB interacts with SLC3A2 to regulate proliferation and tamoxifen resistance in ER+ breast cancer
Source: Commun Biol. 2022 May 2;5:403. doi: 10.1038/s42003-022-03363-3 (PMC9061724; doi:10.1038/s42003-022-03363-3)
Supplement: Supplementary file 6 — Reporting Summary [file 42003_2022_3363_MOESM6_ESM.pdf]

nature portfolio

Corresponding author(s): Yasuhiro Saito, Tomoyoshi Soga, Senthil K. Muthuswamy

Last updated by author(s): Mar 7, 2022

Reporting Summary

Nature Portfolio wishes to improve the reproducibility of the work that we publish. This form provides structure for consistency and transparency in reporting. For further information on Nature Portfolio policies, see our Editorial Policies and the Editorial Policy Checklist.

Statistics

For all statistical analyses, confirm that the following items are present in the figure legend, table legend, main text, or Methods section.

n/a	Confirmed	
☐	☒	The exact sample size (*n*) for each experimental group/condition, given as a discrete number and unit of measurement
☐	☒	A statement on whether measurements were taken from distinct samples or whether the same sample was measured repeatedly
☐	☒	The statistical test(s) used AND whether they are one- or two-sided<br>*Only common tests should be described solely by name; describe more complex techniques in the Methods section.*
☐	☒	A description of all covariates tested
☐	☒	A description of any assumptions or corrections, such as tests of normality and adjustment for multiple comparisons
☐	☒	A full description of the statistical parameters including central tendency (e.g. means) or other basic estimates (e.g. regression coefficient) AND variation (e.g. standard deviation) or associated estimates of uncertainty (e.g. confidence intervals)
☐	☒	For null hypothesis testing, the test statistic (e.g. *F*, *t*, *r*) with confidence intervals, effect sizes, degrees of freedom and *P* value noted<br>*Give P values as exact values whenever suitable.*
☒	☐	For Bayesian analysis, information on the choice of priors and Markov chain Monte Carlo settings
☒	☐	For hierarchical and complex designs, identification of the appropriate level for tests and full reporting of outcomes
☐	☒	Estimates of effect sizes (e.g. Cohen's *d*, Pearson's *r*), indicating how they were calculated

Our web collection on statistics for biologists contains articles on many of the points above.

Software and code

Policy information about availability of computer code

Data collection	no software was used
Data analysis	For quantification of immunoblot signal, we used ImageJ64 software. For survival curve, we used Kaplan-Meier Plotter. For statistic analysis, we used Prism9 (GraphPad software). For metabolomic analysis, we used Metaboanalyst, MassHunter, and MasterHands. For image cropping, we used Photoshop 2021 (Adobe). For ChIP-seq data browse, we used Cistrome database. For Correlation analysis between mRNA expression and gene copy number, we used cBioPortal. For BioID analysis, we used Proteome Discoverer 2.4 software (Thermo Scientific)

For manuscripts utilizing custom algorithms or software that are central to the research but not yet described in published literature, software must be made available to editors and reviewers. We strongly encourage code deposition in a community repository (e.g. GitHub). See the Nature Portfolio guidelines for submitting code & software for further information.

Data

Policy information about availability of data

All manuscripts must include a data availability statement. This statement should provide the following information, where applicable:

- Accession codes, unique identifiers, or web links for publicly available datasets
- A description of any restrictions on data availability
- For clinical datasets or third party data, please ensure that the statement adheres to our policy

The Kaplan-Meier plot data that supports this study's findings are available in Kaplan-Meier Plotter (http://kmplot.com/analysis/) with the identifier (10.18632/oncotarget.10337). The ChIP-seq data “GSM1534722", “GSM808755”, “GSM1010863”, and “GSM1669014” that support the findings of this study are available from Cistrome Data Browser (http://cistrome.org/db/#/). All other relevant data are available from the corresponding author on reasonable request.

Field-specific reporting

Please select the one below that is the best fit for your research. If you are not sure, read the appropriate sections before making your selection.

☒ Life sciences ☐ Behavioural & social sciences ☐ Ecological, evolutionary & environmental sciences

For a reference copy of the document with all sections, see nature.com/documents/nr-reporting-summary-flat.pdf

Life sciences study design

All studies must disclose on these points even when the disclosure is negative.

Sample size	The sample size used in each experiment was not predetermined. Sample sizes were chosen for the different experimental approaches based on the technical difficulty. The chosen sample size are consistent with previous publication.
Data exclusions	No data exclusion
Replication	The experimental findings were reproduced in multiple independent experiments. The number of independent experiments and biological replicates in each data panel is indicated in the figure legends.
Randomization	Randomization was not applicable to our study
Blinding	The investigators were not blinded to group allocation during data collection and analysis

Reporting for specific materials, systems and methods

We require information from authors about some types of materials, experimental systems and methods used in many studies. Here, indicate whether each material, system or method listed is relevant to your study. If you are not sure if a list item applies to your research, read the appropriate section before selecting a response.

Materials & experimental systems

n/a	Involved in the study	
☐	☒	Antibodies
☐	☒	Eukaryotic cell lines
☒	☐	Palaeontology and archaeology
☐	☒	Animals and other organisms
☒	☐	Human research participants
☒	☐	Clinical data
☒	☐	Dual use research of concern

Methods

n/a	Involved in the study	
☒	☐	ChIP-seq
☒	☐	Flow cytometry
☒	☐	MRI-based neuroimaging

Antibodies

Antibodies used	Anti-LAT1 (#5347), anti-4F2hc (#47213), anti-SLC7A11 (#12691), and anti-HA (#3724) were purchased from Cell Signaling. Anti-SCRIB (C-20), anti-SCRIB (C-2), anti-LLGL2 antibody (A-4), anti-CD98 (4F2), anti-CD98 (E-5), anti-Erk2 (C-14), and anti-cyclin D1 (H-295) were purchased from SantaCruz. Anti-HA, anti-CD98hc (BMP090), anti-SLC7A5 (BMP011), and anti-DDDDK-tag (M185) antibodies were purchased from MBL. Anti-SLC7A5 (HPA052673) and anti-SCRIB (HPA023557) antibodies were purchased from ATLAS ANTIBODIES. Anti-Flag antibody (M2) was purchased from Sigma.
Validation	Anti-LAT1 (#5347), anti-4F2hc (#47213), anti-SLC7A11 (#12691), anti-HA (#3724), Anti-SCRIB (C-20), anti-LLGL2 antibody (A-4), anti-CD98 (4F2), anti-CD98 (E-5), anti-HA, anti-CD98hc (BMP090), anti-SLC7A5 (BMP011), and anti-DDDDK-tag (M185), and Flag (M2) antibodies were validated by immunoblot using the cell lysates that overexpress or knockdowned the antigen protein. Anti-SLC7A5 (HPA052673), anti-SCRIB (HPA023557), anti-SCRIB (C-2), anti-Erk2 (C-14), and anti-cyclin D1 (H-295) antibodies were validated by vendors

Eukaryotic cell lines

Policy information about cell lines

Cell line source(s)	BT474, AU565, HCC1569, MCF-7, T47D, MDAMB231 and Hs578T cells were derived from ATCC. Tamoxifen-resistant MCF-7 cell line and the parental MCF-7 cells were kindly provided by Dr. Rachel Schiff from Baylor College of Medicine.
Authentication	None of the cell lines used have been subject to additional authentication

Mycoplasma contamination	All cell lines tested were negative for mycoplasma contamination. Mycoplasma contamination was checked by MycoAlert PLUS (Lonza).
Commonly misidentified lines (See ICLAC register)	No commonly misidentified cell lines.

Animals and other organisms

Policy information about studies involving animals; ARRIVE guidelines recommended for reporting animal research

Laboratory animals	NOD.Cg-Prkdcscid Il2rgtm1Wjl/SzJ, 6 weeks old female mice were purchased from Charles River.
Wild animals	This study did not involve wild animals
Field-collected samples	This study did not involve Field-collected samples
Ethics oversight	All mouse experiments had the approval of the Laboratory Animal Center, Keio University School of Medicine (Protocol #19046-(0)) and were carried out following the 'Guide for the Care and Use of Laboratory Animals'.

Note that full information on the approval of the study protocol must also be provided in the manuscript.
